# Role of traditional healers in psychosocial support in caring for the orphans: A case of Dar-es Salaam City, Tanzania

**DOI:** 10.1186/1746-4269-1-3

**Published:** 2005-07-29

**Authors:** Edmund J Kayombo, Zakaria H Mbwambo, Mariam Massila

**Affiliations:** 1 Institute of Traditional Medicine, Muhimbili University College of Health sciences, P. O. Box 65001, Dar-es-Slaam, Tanzania

**Keywords:** Tanzania, HIV/AIDS, Orphans, Psychosocial support, Traditional healers

## Abstract

Orphans are an increasing problem in developing countries particularly in Africa; due to the HIV/AIDS pandemic; and needs collective effort in intervention processes by including all stakeholders right from the grass roots level. This paper attempts to present the role of traditional healers in psychosocial support for orphan children in Dar-es-Salaam City with special focus on those whose parents have died because of HIV/AIDS. Six traditional healers who were involved in taking care of orphans were visited at their "vilinge" (traditional clinics). In total they had 72 orphans, 31 being boys and 41 being girls with age range from 3 years to 19. It was learned that traditional healers, besides providing remedies for illnesses/diseases of orphans, they also provided other basic needs. Further, they even provided psychosocial support allowing children to cope with orphan hood life with ease. Traditional healers are living within communities at the grass roots level; and appear unnoticed hidden forces, which are involved in taking care of orphans. This role of traditional healers in taking care of orphans needs to be recognised and even scaling it up by empowering them both in financial terms and training in basic skills of psychosocial techniques in how to handle orphans, in order to reduce discrimination and stigmatisation in the communities where they live.

## Introduction

Orphanhood is an increasing problem especially now with the event of HIV/AIDS in developing countries [1-4]. These orphans need, amongst other things, psychosocial support in helping them to cope with orphanhood more easily, by involving all stakeholders right from the grass roots to the national level. Orphans are children who have lost one of their parents or both under eighteen years old [3]. The national data on magnitude of orphans in south of the Saharan African countries is not readily available; and hence the present information is based on estimates. By the end of 2001 it was estimated that 14 million children worldwide had lost their mother or both parents to AIDS or related causes; and it is being projected that by 2010 will reach 35 million [1,5]. Sub-Saharan Africa is the most [2] severely affected region and accounts for more than 80 % of those orphaned as a result of AIDS. In some countries south of Sahara, orphans account for 15–17% of total population [1,5]. In Tanzania, on the otherhand, it is estimated that by the end of 2001 cumulative totals of AIDS orphans were 1.3 (4.3%) million country wide; and the number is expected to rise to 2 million by year 2005 [6].

Much has been written on socio-economic and psychosocial problems encountered by orphans whose parents or guardians have died because of HIV/AIDS [1,3,5-7]. However, major issues are; how does a child deals with a parent's/guardian's loss and what type of care a child receives before and after the death of a parent/guardian and who does that? What is the child's feeling of surviving a parent's/guardian's depression, loss of family income? Furthermore, what is a child's perception of having or not having open communication between family members and/or their community? This calls for skills of psychosocial support to help children cope with orphanhood more easily. Psychosocial support is defined as an ongoing process of meeting physical, emotional, social, mental and spiritual needs of orphans and vulnerable children, all of which are considered to be essential elements for meaningful and positive human development [3]. Family Health International [3] has developed a psychosocial model which can be used in helping children cope with orphanhood. But can this model be applied in a typical traditional setting with increasing numbers of orphans?

It has been argued earlier that Sub-Saharan Africa is the most severely affected region. But who is caring for these orphans in traditional settings? Do they have skills of psychosocial support? It has to be remembered that orphanhood is as old as mankind in history because parents have been dying and living children taken care of by a remaining parent or by elder children, an aunt or uncle or grand parents within the community [1-3,8]. Nevertheless, historical large scale of orphaning has been a sporadic, short term problem associated with war, famine or disease [1]. The word orphan in the African setting was not present. However, in recent years the problem has been aggravated by HIV/AIDS and is increasing; and it is likely to be a long term, chronic problem affecting developing countries through out the world [1,5].

Notwithstanding, studies [1,3,6,8] reviewed still show over 40% of orphans in Sub-Saharan African countries continue to be cared for by traditional structures as was the case in the past and some of them do a good job. For example in Malawi, Alliance [4] has shown how children appreciated the usefulness of their grandmother

*"Our grandmother is so wonderful. She helps us in so many ways. She feeds us, dresses us and brings us up properly. When we see her, we see our mother. If she was not there we would have been scattered around other families and would not be treated in the same way. We are so grateful that she is still with us" *[3]

Catherine 15, the eldest of the eight grand children cared for by Irine 80 years in Malawi

But how many grand parents are doing this wonderful job in the presence of abject poverty in many African communities?

Because of the increasing problem of orphans in some cases orphans are also cared for by elder children. For example, children as young as 7 years are forced to look after their younger brothers and/ or sisters because the extended family no longer has the capacity to absorb more orphans or because the children resist being separated and opt to stay on their own without adult supervision [7]; and this was unusual in the past. The impacts of HIV/AIDS especially in caring for orphans seem to suggest that family structures are changing. Often the middle generation of both men and women are completely absent, leaving old and young to support each other. This means that older members of the family care for the orphans; and orphans and vulnerable children are compelled to take on new roles beyond their years [1].

The questions, which remain unanswered, are; besides giving orphans basic needs can they provide psychosocial support so that these children can cope with orphanhood. We are asking this question because there is evidence that suggests currently there is stigmatisation and in some instances discrimination in food allocation, education and workload from these caregivers [1,7,9]. Is it because of the fear that they can infect their children and caregivers themselves? Or is it the increasing burden of orphan hood in poor families? All in all, the outcome of these problems is that some children leave their relatives and join Community Based Organisations (CBOs) and Non Government organisations (NGOs)[1] especially in urban areas and some go to traditional healers where their parents were being treated. However, we do not know whether it is better to stay at CBOs or NGOs or with the traditional healer as an orphan.

This study used the model of psychosocial support proposed by Family Health International [3] to learn the psychosocial support provided by traditional healers to orphans in an urban setting because there is no literature which attempt to show the role of traditional healers in supporting orphans; and it is one of traditional structures that are involved in healthcare at grass roots level.

The aim of this study was to identify if any: -

- The role of traditional healers in supporting the orphans

- How do they get the orphans?

- The basic needs they can provide to the orphans;

- Techniques used for psychosocial support for orphans;

- The problem encountered when taking the orphans.

## Model of Psychosocial Support

Psychosocial support model is represented with the use of a wheel. The wheel recognizes that none of these elements would be adequate if provided without input from other elements or in a vacuum. If we remove any one segment of a wheel, the wheel would not turn and progress would be impossible. The model assumes that at the centre of the psychosocial wheel model is an awareness of cultural practices, beliefs, and rituals, which inform one about the manner in which all of the other needs are met. Culture needs to be seen as a pivotal point for enrichment of children's identity. Culture also serves as a store of knowledge, values, connectedness, belonging and traditional practice, which is regarded as being essential to the general well being of the child.

One spoke of the wheel would be the physical needs of a child. This incorporates financial needs such as food, shelter, clothing, school uniforms fees and basic health care etc. Most of these economic needs of children are combined with educational needs, for example: the need for information about hygiene, nutritional diet, how to prepare food, decision making models for health care and other skills. The simple provision of financial assistance is not what children need from a psychosocial perspective, but their needs for financial support must be met in an on-going and reliable fashion that also conveys to the child on-going concern, care and support.

A second spoke is emotional needs of children. This includes need for love, security, encouragement, motivation, care, self-esteem, confidence, trust and security, sense of belonging, guidance, understanding etc. Children need to be heard and need to learn to express their feelings in an appropriate manner. At times children's emotional needs may include assisting them to cope with especially difficult circumstances, like bereavement, loss, sexual abuse, etc.

The third spoke is the mental needs, which incorporate three aspects: formal education {schooling}, informal education {opportunities for observational knowledge, adaptation skills, which support the child in order to be able to control environment and access positive reinforcement} and general skills {life skills, general knowledge, etc} combined with motivation and application to succeed.

The fourth spoke would be the children's social needs. These are essential for integration into a community without feeling stigmatised or different; to develop a sense of belonging; form friendships and community ties; acceptance; identity; acknowledgement from peers and opportunities for social interaction. They also need to learn socially acceptable behaviour though feedback from others, how to access help and learn their limits.

The last spoke of the wheel are the children's spiritual needs. Children need a belief, which enables them to develop a hope for their future. They also need to develop trust and security in their survival. This gives them hope to keep trying. Also, this facilitates a sense of connectedness to deceased parents and ancestors. Traditional healers are among the custodians of African traditional culture [10] and therefore the study expected some of the features in caring for the orphans will be manifested in their practice.

## Research Methodology

Dar-es-Salaam is the largest city in Tanzania, and according to 2002 population National Census [11], its growth rate is 4.3% with a total population of 2,497,940 of which 1,236,864 (45.9%) are females living in three Municipalities namely Ilala, Kinondoni and Temeke. Thirty three percent (33%) of the total population are children under 15 years. Notwithstanding, the total number of people living in Dar-es-Salaam today is likely to be higher than mentioned due to rural urban migration and mostly youths coming to seek urban opportunities. In Dar-es-Salaam City, like other cities in developing countries, more than 60% of the population is living in squatter settlements where healthcare and environmental health services are limited.

Dar-es-Salaam City was chosen for this study because; firstly, about one tenth of the total population of Tanzania lives in Dar-es-Salaam. Secondly, the city is composed of almost all 120 ethnic groups of Tanzania; and hence multi-ethnic city and possibly each group in one way or another is captured in this study. Third, Dar-es-Salaam city has many HIV/AIDS patients (ranking third in the nation) and orphans – an area of interest of this study. Fourth, Dar-es-Salaam has NGOs and CBOs which claim to be involved in taking care HIV/AIDS patients and orphan children.

This study is part of Traditional Medicine HIV/AIDS project, which started in 2003 September and is ongoing still to date. The Traditional Medicine HIV/AIDS project recruited 25 traditional healers. The selection of these traditional healers was based on their reputation in providing healthcare to HIV/AIDS patients and also from records of traditional healers associations. The research team visited selected traditional healers at their respective vilinge. It was during these visits the research team learned that six traditional healers were caring for orphans. The research team was interested to learn how they got these orphans; and ways of caring for orphans in traditional settings and problems encountered. Further, specifically focus was on psychosocial support offered to these children.

As shown earlier the psychosocial model proposed by Family Health International [3] was used in this study. An in-depth interview questionnaire in Kiswahili language was developed as a research instrument for interviewing traditional healers who were caring for orphans whose parents had died because of HIV/AIDS. Kiswahili is the national language of Tanzania; and most of urban people are well versed with it as a means of communication. The questionnaire focused on their role of taking care of orphans and seeing how they fit in to the psychosocial model like how they got them, what things did they offer to orphans so that they can cope with orphanhood, techniques of psychosocial support (if any) and problems encountered when taking care of orphans. The research team itself with the use of notes and a tape recorder did the data collection. The collected data were screened and then transcribed from Kiswahili to English. The data collected were summarized and codes were identified for grouping the information according to issues raised in the introduction. The grouped information per code were re-summarized for getting the finally report. The summary of the final report of findings is presented below.

## Results

### The role of traditional healers in supporting the orphans

The study findings show that traditional healers were not only treating patients using traditional remedies, but also involved in caring for orphans using traditional psychosocial support by providing with them the basic needs and psychosocial support as shown in the psychosocial support model. Out of 25 traditional healers who were in the Traditional Medicine HIV/AIDS Project in first phase, 6 (24%) were involved as well in taking care of orphans. Five of these traditional healers were males aged between 35–50 years, and one female aged 46 years. The total number of orphans recorded from these traditional healers in study period, whose parents died because of HIV/AIDS, was 72 children ranging from 3–19 years. There distribution by age and sex is shown in Table 1. Most of these orphans (38.9%) were at age of 5–9, followed by age 15–19 (26.4%).

**Table 1:** Distribution of Orphans Who are Under the Care of Traditional Healers by Age Group and Sex

Age group	Sex		Total
	male	females	

0–4	6 (8.3%)	5 (6.9%)	11(15.2%)
5–9	10 (13.9%)	18 (25%)	28 (38.9%)
10–14	6 (8.3%)	8 (11.1%)	14 (19.4%)
15–19	9 (12.5%)	10 (13.9%)	19 (26.4%)
Total	31 (43%)	41 (56.7%)	72 (100%)

Three traditional healers expressed that some of children were denied by relatives after death of all parents for fear that these children may infect their children through playing or exchange of things they eat like sweets, sweet potatoes or cassava that are being sold along the streets. Further, these children were abused in words by their respective relatives that their parents died of AIDS because of their bad behavior or promiscuity. All these together lead children to move away and in this way reach traditional healers, CBOs and NGOs and some end up on street.

Table 2, on the other hand, shows that 32 (44.4%) of these orphans had lost their fathers, 25 (34.7%) mothers and 15 (20.8%) both parents. Above twenty-seven percent (27.8%) had been screened and found to be HIV positive; and were between the age of 15–19 years.

**Table 2:** Number of orphans by kind of parents they have lost

Number of children By sex	Kind of parent who has died			Total
	father	mother	Both parents	

male	15 (20.8%)	11 (15.3%)	7 (9.7%)	33 (45.8%)
Female	17 (23.6%)	14 (19.4%	8 (11.1%)	39 (54.2%)
Total	32 (44.4%)	25 (34.7%)	15 (20.8%)	72 (100%)

The Secretary General of Tanzania Traditional Health Practitioners Association (TATHEPA) reported that "We are getting these children always and we have no where to send them because of their extended family generally lives far away from Dar- es -Salaam; and we have decided to stay with them as part of our family; and our main role as traditional healers is to support them in whatever we can so that they can cope with orphan hood; and where possible we attempt to visit relatives and have a discussion of how to assist these orphans".

### How did they get the orphans?

The study explored ways in which traditional healers got orphans whom they were caring for and found out that they obtained them in three ways. First, some of orphans were from traditional healers' relatives and were asked to look after them after the death of parents as it is in traditional African culture. Two, other orphan children were from neighbours who came for treatment after being infected with HIV/AIDS. Children from neighbours lived at their respective relatives but during the day came to play at traditional healers' compounds as a place for socialising with other children. It was here where sometimes these orphans got their lunch and even dinner. Three, some of the orphans were abandoned by relatives at traditional healers' *vilinge *as they came to seek healthcare; and hence these healers have to accommodate them as part of their families.

During interviews traditional healers reported that while caring for these orphan children common problems observed from these children were repeated illness especially those who were HIV positive, anger, guilt conscience, fear, sometimes isolation from playing mates and feeling that they were being oppressed. Further, it was reported that some of the children were crying now and then especially young ones "*baba yangu yuko wapi *"(where is my father) or *mama yangu yuko wapi *(where is my mother), sleepless nights and nightmares were common. Several attempts to fulfill both basic needs and psychosocial supports were made to comfort them.

### The basic needs Traditional healers can provide to orphans

Even though traditional healthcare practitioners do not earn much money in traditional medicine practice, the research team from this study learned that traditional healers attempted to provide basic needs to orphans who were under their control to help them cope with orphanhood life in their early stage of life. For example, the traditional healers in this study reported having given many things to these children in the process of making them feel at home and cope with orphanhood. Among them were basic needs like food, clothes, school uniforms and exercise books, etc for those who were in school; medical care and safety by making them part of their families. They ate together and allowed them to play with their children; and hence fitting well in first spoke and fourth spoke of the wheel in the psychosocial support model. Nevertheless these traditional healers encountered some problems as stressed by one traditional healer that

"we do not meet all what these children want to live happily because of our poor economic situation. Some of the children stay with us for a while and leave if we fail to meet their demands. Some of these children join street children we see in Dar"

### Techniques used for psychosocial support of orphans

During interviews on traditional healers' practices the research team noted they have different traditional methods that are useful for helping orphans cope with orphanhood and live more easily. For example, three traditional healers reported that when children face problems like crying, talking at night were signs that they were seeing "shadows" of their respective parents and grand parents who have died; and had to give them remedies that can remove the parents' and grand parents' "*shadows*". Remedies may be something to wear either on the hand or around the neck. Whereas, two traditional healers, reported that they had to bath children with herbal remedies. The remedies were vitalised by words of healers through short litany of prayer asking parents and grand parents by naming them to leave the child so that s/he can stay comfortable. In addition, they comforted the orphans by telling stories in the evening; and citing several examples of orphans who have managed to cope with orphan hood and had achievement in life. All these activities reported in this section fits best second and third spoke of the wheel of psychosocial support model. All in all these symbols were meant to help children to forget what happened in the past and build a positive attitude towards living as orphans.

Besides the above, while the research team was visiting vilinge of traditional healers, they had an interesting experience where one of six traditional healers interviewed had 54 orphans. Of these 31 (57.4%) were girls and 23 (42.6%) being boys. Eleven (20.4%) of these have lost both parents, and 30 (55.5%) have lost their fathers and rest have lost their mothers. Eight (14.8%) of these children were HIV/AIDS positive.

This healer reported that she developed an interest in taking care of orphans after loosing her two bothers that left her with seven children to look after according to the African traditional culture. While practicing traditional medicine to HIV/AIDS patients she felt that orphans had a desperate life. She decided to collect other orphan children from her community and provided with them activities which help them to forget the past; and they were basically geared to psychosocial support as shown in the psychosocial model. For example, the healer involved the orphans in poetic theatre, theatre; drama, choir, whereas the others dealt in comics and traditional dances. These groups met twice a week for practice and performed when invited to a social gathering function where they received payment. The poetic theatre group has managed to record one single CD. The healer stressed, "These activities help orphans to forget the past and build a new outlook"

### The problem encountered when taking the orphans

Despite traditional healers efforts to help orphans to cope with orphanhood they faced several challenges which need government and NGO support. The common challenges reported by all six traditional healers interviewed were: firstly, limited resources to meet children's basic needs and make them live happily. Secondly, repeated illness especially those who were HIV positive and health care for health problems which can be managed effectively in formal health services. Thirdly was the accommodation particularly for healers who have rented rooms for their families. Fourth was the inadequate food for feeding surplus numbers of people in their families. Finally tracing back relatives for orphans being abandoned at traditional healers' vilinge *when *these children become very seriously sick.

## Discussion

The findings from this present study shows that traditional healers have a significant role in caring for orphans in communities using a holistic approach even though they are hardly reported in the literature. Grand parents who are caring for orphans appear to be overwhelmed with the orphans, leading them to sell even their assets or borrow money to meet daily needs at home [2] because of abject poverty. Again many NGOs and CBOs that are involved in taking care of orphans in Tanzania [6] and elsewhere in developing countries follow strategies of traditional methods of focusing on basic needs like food, clothing, shelter and medical care and going to school [1]. But caring for orphans needs to go beyond this by taking consideration of psychosocial support that helps these children to cope with life. It has to be remembered that death of parents affects children socially and psychologically and has a great impact on the orphans in daily life [7,12,13]. People caring for orphans need to capture this aspect as well to make them cope with life more easily.

In the present study, the analysis shows that traditional healers use a holistic approach when handling orphans by taking into account both basic needs and psychosocial support to make a whole support package. The analysis of traditional healers' activities when caring orphans, underscores The International Federation of Red Cross and Red Crescent Societies [13] that key components of psychosocial support for orphans are ongoing interaction and presence with the aim of bolstering feelings of security and hope and eventually attempting to meet nearly all essential components in each of the spokes of psychosocial model. This support endeavours to help the affected orphans strengthen their personal coping capacities, hope, security, trust and reinforce support from family members and friends [3,7].

Furthermore, the analysis from the traditional healers interviews supports the Family Health International [3] proposed model on psychosocial support in caring orphans on the role of culture as pivotal. The "shadows" from parents and grand parents who have died reflects the belief of traditional African culture that people who have died are alive in an "other world" and have an influence on people who are living in our world; and that is why they believed that they come in the form of "shadows" to their beloved ones. To remove the "shadows" is done through rituals which are accompanied with litany of names of people who have died asking them to stop coming to orphans because they create fear and make them fail to cope with orphanhood [14]. Moreover, the analysis from traditional healers' findings appears to cement the psychosocial support model argument that culture needs to be seen as a pivotal point for the enrichment of children's identity and coping with orphan hood.

Besides the above, traditional healers are becoming creative; and without knowing found themselves doing psychcosocial activities with orphans. For instance, the art comforting orphans like giving them living examples for some of people who have lost their parents and managed to make there way through; and story telling in the evening reduces time of thinking of the past. Also healer who collected the orphans and engaged them in social activities like poetic theatre, theatre comics, traditional dances, had strong impact on psychosocial support to these children. These activities help children to integrate into a community without feeling stigmatised or different. Also, these activities help to develop a sense of belonging, form friendships and community ties. Above, all these things help to develop acceptance; identity; acknowledgement from peers and opportunities for social interaction, trust, security and hope for the future.

Traditional healers do not only end up in psychosocial support of orphans but also provided materials like foods, clothes, and exercise books and allowed them to play with their children; where necessary they even provided bus fares to schools. All these aspects make them think that they are also being valued. These supports fit in the other spokes of the wheel of Family Health International's [3] proposed model on psychosocial support. However, as shown in the findings these traditional healers cannot meet all basic needs for orphans because they do not have enough financial resources. Financial resources they use are fees collected from patients, which are again limited.

All in all the findings in this study seem to suggest that traditional healers are a hidden force unnoticed in taking care of orphans in communities. With increasing number of orphans due to HIV/AIDS, this experience suggests that orphan children are best cared for within families and communities from the same cultural milieu including traditional healers. However, this needs creativity and empowerment of community structures that are involved in caring for orphans by giving them basic training on psychosocial techniques of how to handle orphans as stipulated in Family Health International's [3] proposed model on psychosocial support. This can be done by involving community groups like faith based congregations, and CBOs and NGOs who are first in line for households caring for vulnerable children [3]. Emphasis should be on psychological support which will help children to cope with orphan hood like sports, poetic theatre, setting income generating activities, providing spiritual, emotional and psychological support. In addition the government should support these types of healers through credit schemes.
